# Retraction: MiR-424-5p Inhibits Proliferation, Invasion and Promotes Apoptosis and Predicts Good Prognosis in Glioma by Directly Targeting BFAR

**DOI:** 10.3389/pore.2021.1609970

**Published:** 2021-08-23

**Authors:** Pathology & Oncology Research Editorial Office

**Affiliations:** Frontiers Media SA, Lausanne, Switzerland

The journal and authors retract the article cited above for the following reasons provided by the authors:

The authors state “We cannot repeat some of the biological results of this article, which directly leads to our inability to carry out follow-up research. Based on the above objective reasons, after careful discussion and consideration, our team decided to withdraw the manuscript in a rigorous and scientific attitude”.

This retraction, as requested by the authors, was approved by the Editors-in-Chief of the Journal.

